# Associations of the plasma lipidome with mortality in the acute respiratory distress syndrome: a longitudinal cohort study

**DOI:** 10.1186/s12931-018-0758-3

**Published:** 2018-04-10

**Authors:** Michael D. Maile, Theodore J. Standiford, Milo C. Engoren, Kathleen A. Stringer, Elizabeth S. Jewell, Thekkelnaycke M. Rajendiran, Tanu Soni, Charles F. Burant

**Affiliations:** 1 Department of Anesthesiology, Division of Critical Care Medicine, University of Michigan Medical School, 4172 Cardiovascular Center, 1500 East Medical Center Drive, SPC 5861, Ann Arbor, MI 48109 USA; 2 Department of Internal Medicine, Division of Pulmonary and Critical Care Medicine, University of Michigan Medical School, Ann Arbor, Michigan USA; 3 Department of Clinical Pharmacy, College of Pharmacy, University of Michigan, Ann Arbor, Michigan USA; 4 Michigan Center for Integrative Research in Critical Care, University of Michigan, Ann Arbor, Michigan USA; 5 Michigan Center for Translational Pathology, Department of Pathology, University of Michigan, Ann Arbor, Michigan USA; 6 Michigan Regional Comprehensive Metabolomics Resource Core, University of Michigan, Ann Arbor, Michigan USA; 7 Division of Metabolism, Endocrinology, and Diabetes, University of Michigan, Ann Arbor, MI USA

**Keywords:** Acute respiratory distress syndrome, Lipids, Metabolomics, Critical care, Hospital mortality

## Abstract

**Background:**

It is unknown if the plasma lipidome is a useful tool for improving our understanding of the acute respiratory distress syndrome (ARDS). Therefore, we measured the plasma lipidome of individuals with ARDS at two time-points to determine if changes in the plasma lipidome distinguished survivors from non-survivors. We hypothesized that both the absolute concentration and change in concentration over time of plasma lipids are associated with 28-day mortality in this population.

**Methods:**

Samples for this longitudinal observational cohort study were collected at multiple tertiary-care academic medical centers as part of a previous multicenter clinical trial. A mass spectrometry shot-gun lipidomic assay was used to quantify the lipidome in plasma samples from 30 individuals. Samples from two different days were analyzed for each subject. After removing lipids with a coefficient of variation > 30%, differences between cohorts were identified using repeated measures analysis of variance. The false discovery rate was used to adjust for multiple comparisons. Relationships between significant compounds were explored using hierarchical clustering of the Pearson correlation coefficients and the magnitude of these relationships was described using receiver operating characteristic curves.

**Results:**

The mass spectrometry assay reliably measured 359 lipids. After adjusting for multiple comparisons, 90 compounds differed between survivors and non-survivors. Survivors had higher levels for each of these lipids except for five membrane lipids. Glycerolipids, particularly those containing polyunsaturated fatty acid side-chains, represented many of the lipids with higher concentrations in survivors. The change in lipid concentration over time did not differ between survivors and non-survivors.

**Conclusions:**

The concentration of multiple plasma lipids is associated with mortality in this group of critically ill patients with ARDS. Absolute lipid levels provided more information than the change in concentration over time. These findings support future research aimed at integrating lipidomics into critical care medicine.

**Electronic supplementary material:**

The online version of this article (10.1186/s12931-018-0758-3) contains supplementary material, which is available to authorized users.

## Background

Despite years of research, the mechanisms by which ARDS leads to multiple organ failure and death remains poorly understood. While general improvements in ventilator management and critical care medicine have improved outcomes, no targeted therapies exist. To date, all treatments designed to interrupt progression have failed in clinical trials. This may represent an inadequate recognition of different phenotypes that exist within ARDS or an incomplete understanding of relevant biologic pathways.

Metabolomics, the measurement of metabolites in a biological system, can help with this by providing a fingerprint of the biochemical milieu of affected patients. This information will help find new biomarkers, develop new therapeutic targets, and improve our ability to personalize treatment. Several studies support this notion in both critically ill adult [1–3] and pediatric [4] patients, with some of these studies suggesting that carbohydrate and amino acid metabolism change early during the inflammatory response followed alterations in the lipidome [5].

Lipid biology likely plays an important role in the pathophysiology of ARDS. For example, tumor necrosis factor-α increases metabolism of arachidonic acid by cyclooxygenase, which generates the mediators responsible for increased inflammation. A shift to metabolism by lipoxygenase helps to terminate this process [6–8]. Knock-out models of both cyclooxygenases and lipoxygenases support the important role of lipid biology in the inflammatory response by showing that deficiencies in cyclooxygenase-2 lead to increased mortality in sepsis [9], while decreased 5-lipoxygenase had the opposite effect [10]. Most metabolomic studies to date focus on water-soluble metabolites. Additional research needed to identify lipids with important roles in the pathophysiology of critical illness.

To evaluate the informativeness of the plasma lipidome in ARDS, we performed an analysis of samples collected in a previous trial (ClinicalTrials.gov identifier: NCT00201409). This previous trial examined the utility of recombinant human granulocyte-macrophage colony stimulated factor (GM-CSF) for patients with acute lung injury based on pre-clinical data suggesting a protective effect of GM-CSF on alveolar macrophages and alveolar epithelial cells [11]. The results demonstrated no improvement with GM-CSF treatment. We hypothesized that both the absolute concentration and change in concentration over time of plasma lipids are associated with 28-day mortality.

## Methods

### Study population

The Institutional Review Board at the University of Michigan approved this study. Samples were previously collected as part of a multi-centered prospective clinical trial examining the utility of GM-CSF for critically ill adult patients with ARDS. Research funds allowed for the analysis of 60 samples. Therefore, 30 subjects with samples available from two time points were selected to maximize sequential organ failure assessment (SOFA) scores. Two time points were analyzed in order to compare the importance of the change in lipid concentrations over time with the absolute lipid concentrations measured early in the course of disease. The details of the original study, which randomized patients with acute lung injury or ARDS to receive GM-CSF or placebo, have been published [11]. The definition of ARDS was consistent with guidelines that were available at the time of the original study [12]. Exclusion criteria for the original study included evidence of preexisting chronic respiratory failure, neutropenia (absolute neutrophil count < 1000 cells/mm^3^), and a history of hematologic malignancy or bone marrow transplantation. Ventilator management followed a protocol that focused on minimizing tidal volumes and plateau pressures [13].

### Sample preparation

Plasma samples were stored at − 80 °C until the time of analysis, which was conducted by the Michigan Regional Comprehensive Metabolomics Resource Core. Lipids were extracted using a modified Bligh-Dyer Method [14]. The extraction was performed using water/methanol/dichloromethane (2:2:2 *v*/v/v) at room temperature after the addition of internal standards. The organic layer was then collected and dried under a stream of nitrogen before being re-suspended in 100 μL of Buffer B (5%H_2_O:10%ACN:85%IPA containing 10 mM NH_4_OAc) and analyzed using a liquid chromatography tandem mass spectrometry (LC/MS/MS) lipidomics assay [15].

### Liquid chromatography/mass spectrometry

The lipid extract was injected onto a 1.8 μm particle 50 × 2.1 mm internal diameter Waters Acquity HSS T3 column (Waters, Milford, MA) that was heated to 55 °C. For chromatographic elution, we used a linear gradient over a 20 min total run time. A 60% Solvent A and 40% Solvent B gradient was used for the first 10 min. Then the gradient was ramped in a linear fashion to 100% Solvent B which was maintained for 7 min. Thereafter, the system was switched back to 60% Solvent B and 40% Solvent A for 3 min. The flow rate used for these experiments was 0.4 mL/min and the injection volume was 5 μL. The column was equilibrated for 3 min before the next injection and run at a flow rate of 0.400uL/min for a total run time of 20 min. Data were acquired in positive and negative modes using data-dependent MS/MS with dynamic mass exclusion. Pooled human plasma samples and pooled experimental samples (prepared by combining small aliquots of each experimental sample) were used to control for the quality of sample preparation and analysis [16]. A randomization scheme was used to distribute pooled samples within the set and a mixture of pure authentic standards was used to monitor instrument performance on a regular basis.

### Lipid identification

Lipids were identified using the LIPIDBLAST computer-generated tandem MS library [17]. This database contains 212,516 spectra covering 119,200 compounds representing 26 lipid classes, including phospholipids, glycerolipids, bacterial lipoglycan, and plant glycolipids. Quantification of lipids was completed using AB-SCIEX MultiQuant software. After excluding compounds with a relative standard deviation greater than 30% in the pooled samples, the raw mass spectrometry data were normalized using the internal standards and adjusted for batch effects using loess smoothing. The nomenclature used for individual lipids begins with the abbreviation of the lipid class (Table 1) followed by the number of carbon atoms in the molecule and then by the number of double bonds. If the same lipid was detected in both the positive and negative modes, an underscore after the name of the lipid was used to denote the second measurement.

**Table 1:** Lipid classes included in this study and their abbreviations

Lipid Class	Abbreviation
Phosphatidylcholine	PC
Lysophosphatidylcholine	lysoPC
Plasmenyl Phosphatidylcholine	plasmenyl-PC
Phosphatidylethanolamine	PE
Lysophosphatidylethanolamine	lysoPE
Plasmenyl Phosphatidylethanolamine	plasmenyl-PE
Sphingomyelin	SM
Phosphatidic acid	PA
Phosphatidylinositol	PI
Phosphatidylglycerol	PG
Cardiolipin	CL
Cholesteryl ester	CE
Monoacylglycerol	MG
Diacylglycerol	DG
Triacylglycerol	TG

### Statistical analysis

We removed lipids with coefficients of variation > 30% to focus on more precisely measured compounds. The remaining concentrations were log transformed and auto scaled (mean-centered and divided by the standard deviation of each variable) to create normally distributed lipid concentrations and make the lipid concentrations more comparable to each other, respectively. To ensure that the administration of GM-CSF did not impact lipid concentrations, the lipid levels were compared between those did and did not receive the study drug. Next, the differences in lipid concentrations between survivors and non-survivors were compared using repeated measures analysis of variance. Lipid concentrations were the independent variable and mortality was the dependent variable. False discovery rate (FDR) adjusted *p*-values (q-values) were used to account for multiple comparisons [18]. A q-value less than 5% was considered statistically significant. Heatmaps were used to display both the relative concentrations of significant lipids and the Pearson product-moment correlation coefficient (PPMCC) between significant lipids. The PPMCC was calculated separately for each combination of lipids analyzed. Scatterplots of each lipid relationship were examined to identify the presence of nonlinear relationships. Hierarchical clustering was performed using Pearson distance and the complete linkage clustering algorithm. The overall differences in the variability and distribution of lipid concentrations between survivors and non-survivors was evaluated using principal component analysis (PCA). Finally, in order to describe associations between lipid concentrations and outcomes, the area under the receiver operating characteristic (AUROC) for the lipid concentrations at the first time point were compared with the Acute Physiology Score (APS) from the Acute Physiology and Chronic Health Evaluation III [19] at randomization and the SOFA [20] score obtained at the time of sample collection. Software used in the analysis of these data were MetaboAnalyst 3.0 (Xia Lab, McGill University, Montreal, Canada) [21], SAS software (Version 9.4, SAS System for Windows, SAS Institute Inc., Cary, NC, USA), and R (version 3.2.1; R Foundation, Vienna, Austria).

## Results

The study population consisted of 30 critically-ill subjects with ARDS who each had samples analyzed from two different days (Table 2). This group contained a similar number of males (53%) and females (47%) and was almost entirely white. Most subjects received nutritional support on the days that samples were collected; only five did not. The original study did not collect details about the type or amount of nutritional support received by participants.

**Table 2:** Subject characteristics at enrollment

Characteristics	Alive (*n* = 22)	Dead (*n* = 8)
	Median (Q1,Q3)	Median (Q1,Q3)
Age (years)	48 (36, 54)	41 (39.5, 66)
SOFA Score	5 (4, 7)	7 (4.5, 9)
APS Score	52.5 (41.0, 61.0)	67.5 (51.5, 71.0)
	n (%)	n (%)
Diagnosis		
Pneumonia	9 (40.9)	3 (37.5)
Sepsis	8 (36.4)	3 (37.5)
Aspiration	2 (9.1)	1 (4.6)
Pancreatitis	0 (0.0)	1 (4.6)
Postoperative	1 (4.6)	0 (0.0)
Emergent Surgery	1 (4.6)	0 (0.0)
Other	1 (4.6)	0 (0.0)
Male	12 (54.6)	4 (50.0)
Race		
White	20 (90.9)	8 (100.0)
Black	1 (4.6)	0 (0.0)
Other	1 (4.6)	0 (0.0)

*APS* Acute Physiology Score, *ARDS* Acute Respiratory Distress Syndrome, *Q1* First Quartile, *Q3* Third Quartile, *SOFA* Sequential Organ Failure Assessment

Three hundred fifty-nine compounds remained after eliminating those with a high coefficient of variation. Those remaining consisted of lipids from four of the eight lipid categories described by the LIPID MAPS consortium (glycerolipids, glycerophospholipids, sphingolipids, and sterol lipids). No significant associations existed between subjects receiving and not receiving GM-CSF. Therefore, all 359 lipids were included in the subsequent analysis of associations between the plasma lipidome and mortality in ARDS.

**Fig. 1:**
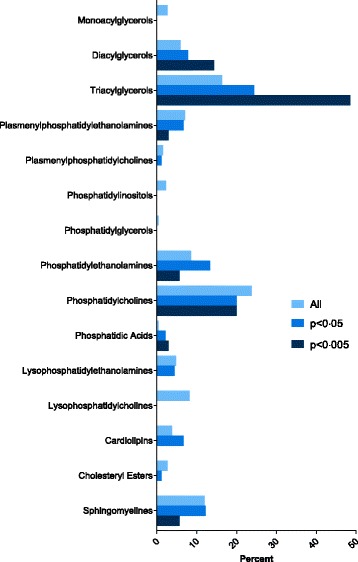
Composition of the lipidome for all lipids, those that differ between cohorts at a significance level of 0.05, and those that differ between cohorts at a significance level of 0.005. This reveals that diacylglycerols, triacylglycerols and phosphatidic acids represent a greater proportion of the total as the significance level is increased, which demonstrates that the lipids most strongly associated with mortality do not mirror the composition of the lipidome. The percentages represented by the bars sum to 100% for each of the criteria

Ninety lipids (25% of all lipids studied) differed between survivors and non-survivors after adjusting for multiple comparisons (q > 0.05). Given the large number of compounds, we increased the significance level several times to identify the compounds that were most consistently altered in those with 28-day mortality (Fig. 1). This revealed strong associations between mortality and altered concentrations of lipids within the DG, TG, and PA classes.

**Fig. 2:**
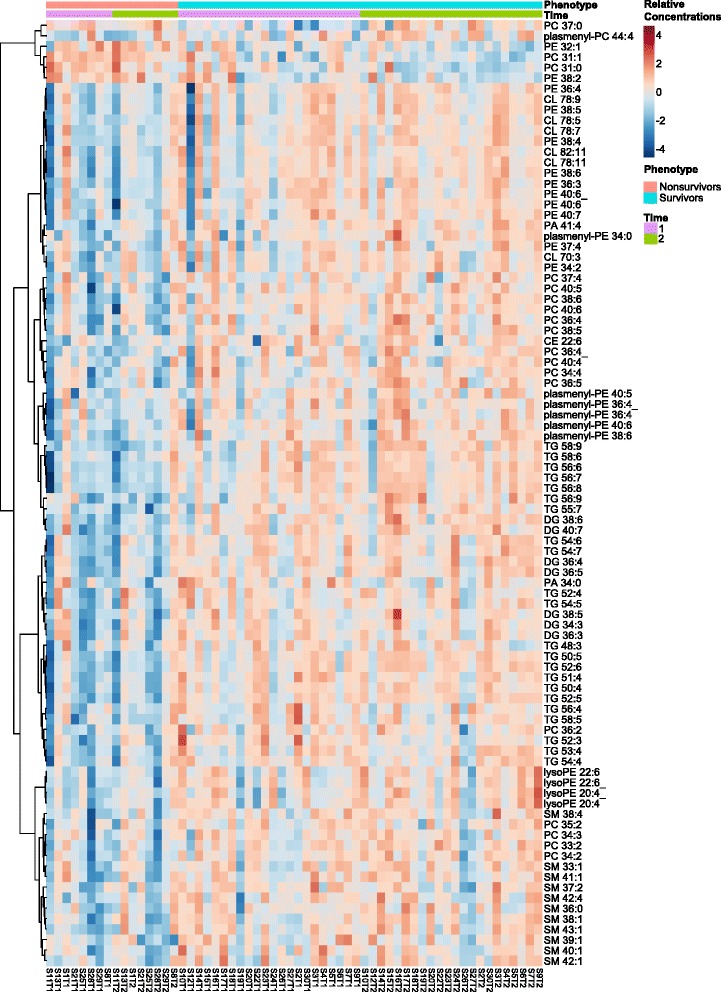
Heatmap of the relative concentrations of each of the 90 lipids that differed between survivors and nonsurvivors. Measurements are separated according to the outcome of the subject and the time point. Overall, the concentration of most lipids (85 of 90) are greater in survivors compared to nonsurvivors. No pattern is appreciated between the two time points. Average values for the two cohorts can be found in Additional file 1: Table S1. Lipids are arranged based on hierarchical clustering using a complete linkage algorithm. The dendrogram shows how lipids were arranged relative to each other. The labels at the bottom of the figure show the subject number and time point

While the concentration of multiple lipids differed between cohorts, no association existed between mortality and the change in concentration over time. The relationship between mortality and both the absolute concentration and the change in concentrations is visualized using a heatmap (Fig. 2). Regarding the absolute lipid concentration, almost all levels were higher in survivors. Only five membrane lipids (PE 32:1 and 38:2 and PC 31:1, 31:0, and 37:0) had the opposite relationship. Regarding the intrasubject change in lipid concentration over time, no associations existed between the within subject lipid concentration change over time. The table located in Additional file 1 contains the values that correspond to the colors in the heatmap.

To explore the relationship of the 90 significant lipids with each other, we created separate correlation matrices for survivors and non-survivors and then calculated the difference in the correlation coefficients between these cohorts. We generated a heatmap based on these differences and used hierarchical clustering to group lipids per the group difference in correlations (Additional file 2). This matrix displays information from the first time point of all 30 subjects across 90 lipids and reveals which lipids have a greater correlation in survivors or non-survivors and provides insights into the inter-lipid relationships that are important for each cohort. Four of the five lipids with greater concentrations in non-survivors were clustered together and tended to have greater correlation with other lipids in the cohort that survived. In other words, for non-survivors, we observed an altered relationship in this group of lipids. Clusters of lipids that showed the greatest increase in correlation among survivors and were overall higher in concentration contained many glycerolipids composed of polyunsaturated fatty acids (PUFAs).

**Fig. 3:**
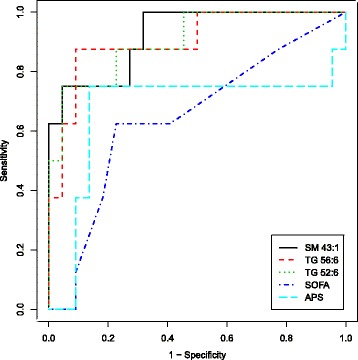
Area under the receiver operating characterstic curves for the three lipids with the best discrimination between survivors and non-survivors. Two commonly used severity of illness scores are also displayed for comparison. Many plasma lipids, including the one sphingomyelin and two triacylglycerol species displayed, outperformed the sequential organ failure assessement score and the acute physiology score

Finally, to further investigate the associations between lipid concentrations and outcome, we calculated the AUROC for the 90 significant lipids, the APS at randomization, and the SOFA score at the time the sample was obtained (Additional file 3). Most of the lipids that differed between survivors and non-survivors had better discrimination than both the APS (AUROC = 0.67) and SOFA score (AUROC = 0.64). Three lipids (SM 43:1, TG 56:6, TG 52:6, and TG 52:5) had excellent discrimination with each having an AUROC > 0.9 (Fig. 3).

## Discussion

This study found multiple associations between the plasma lipidome and ARDS mortality. Ninety lipids differed between survivors and non-survivors when using a relatively strict level of significance for exploratory research (q < 0.05). Except for five compounds, lipids differing between groups had a higher concentration in survivors. These findings are consistent with those published by Ferrario et al., who reported a similar relationship in a study of septic shock [22]. Together, these studies suggest that reduced lipid substrate underlies poor outcomes in critical illness. Given the decreased concentrations of PUFA containing glycerolipids in non-survivors, inadequate substrate for energy production or generation of mediators may drive part of the poor outcome. Unlike the study by Ferrario et al. [22], we did not find a relationship between mortality and the change in lipid levels over time. Explanations for this difference include the lower severity of illness found or more stringent significance level used in our study.

A large body of evidence exists pertaining to the importance of PUFAs for ARDS mortality. Previous guidelines even recommended the administration of fish oil (source of the omega-3 eicosapentaenoic acid and docosahexaenoic acid) and borage oil (source of the omega-6 fatty acid linolenic acid) to individuals with ARDS [23–27]. These PUFAs were thought to reduce the inflammatory process that led to progressive lung injury. This practice decreased after the OMEGA trial showed no difference with treatment and possible harm [28], although some still advocate for this practice [29]. A potential reason for the failure of this therapy is a failure to correctly distinguish which individuals would benefit. Our results raise the possibility that the plasma lipidome provides a tool that may identify a subset of ARDS patients who would benefit from lipid supplementation with certain lipids. Inadequate lipid substrate may prevent a critically ill individual from meeting the increased metabolic demands, mounting an adequate inflammatory response, or generating the mediators necessary to terminate the inflammatory response. Conversely, providing excess substrate to individuals with an adequate supply may overwhelm regulatory processes leading to unnecessary inflammatory or anti-inflammatory responses.

While most lipids were decreased in non-survivors, several were not. We found increased concentrations of one sphingomyelin, two phosphatidylcholines, and two phosphatidylethanolamines compounds in this population (Fig. 2, Additional file 1). These also showed greater correlation with other significant lipids in survivors compared to non-survivors (Additional file 2). A similar relationship has been described before for phosphatidylcholine [30] and sphingomyelin [22] while our findings for phosphatidylethanolamine are novel. The importance of these compounds may relate to their involvement in the coagulation system. For example, phosphatidylethanolamine increases the inactivation of factor Va by activated protein C when added to phospholipid vesicles [31] and sphingomyelin is involved in the regulation of thrombin generation [32]. Therefore, release of these membrane lipids into the circulation may mediate some of the coagulation disorders seen in critical illness [33]. The use of lipid assays to identify affected individuals may help to isolate individuals that would benefit from therapies such as activated protein C [34, 35], which failed to improve outcomes when administered to an untargeted group of patients [36, 37].

Our findings must be tempered by the hypothesis-generating nature of this work and several limitations should be considered when interpreting these results. First, since this study contained a small number of subjects relative to the number of lipid mediators, we were not able to perform post-hoc analyses aimed at understanding the mechanisms underlying the observed differences. While adjustments were made for multiple comparisons, we had inadequate power to adjust for the confounders such as propofol administration. Propofol contains both soybean oil and egg lecithin, which contain acylglycerols with fatty acids from the phosphatidylcholine, phosphatidylethanolamine, and phosphatidylinositol classes [38]. Both lipids that were increased and decreased in survivors could have been affected and future research is needed to understand if lipid containing medications affect the patients in ways other than their primary pharmacodynamic effect. Second, this study did not determine the location of double bonds present in fatty acids. It is known that n-3 and n-6 fatty acids affect the inflammatory response in different ways, but our analysis was not able to determine the proportion of fatty acids of each type. Finally, the participants of this study were almost entirely white and findings may not apply to other races.

## Conclusions

These findings suggest that the plasma lipidome of patients with ARDS may identify those at high risk for mortality. If this is confirmed in subsequent studies, lipidomic profiles may allow us to more accurately determine who will benefit from certain treatments and move us toward personalized care of critically ill patients.

## Additional files

Additional file 1: **Table S1.** Relative lipid concentrations ranked according to the difference between cohorts. (DOCX 52 kb)

Additional file 2: Heatmaps of the differences in the Pearson’s r correlation coefficient between survivors and nonsurvivors. Lipids are arranged based on hierarchical clustering using a complete linkage algorithm. The dendrogram shows how lipids were arranged relative each other. The group of lipids that has a greater correlation in survivors (red font) contains most of the lipids that had a greater concentration in those who died and contains many lipids that originate from the cell membrane. The cluster with the greatest amount of correlation among non-survivors (yellow highlighting) contains primarily glycerolipids consisting mostly of polyunsaturated fatty acids. These observations suggest that these groups of lipids play an important role for the outcome of patients with ARDS. (PDF 119 kb)

Additional file 3: **Table S2.** Area under the receiver operating characteristic curve for lipids that differ between survivors and non-survivors. (DOCX 35 kb)

## Abbreviations

APS: Acute physiology score
ARDS: Acute respiratory distress syndrome
AUROC: Area under the receiver operating characteristic curve
CE: Cholesteryl ester
CL: Cardiolipin
DG: Diacylglycerol
FDR: False discovery rate
GM-CSF: Granulocyte-macrophage colony stimulated factor
lysoPC: lysophosphatidylcholine
lysoPE: lysophosphatidylethanolamine
MG: Monoacylglycerol
PA: Phosphatidic acid
PC: Phosphatidylcholine
PE: Phosphatidylethanolamine
PG: Phosphatidylglycerol
PI: Phosphatidylinositol
plasmenyl-PC: plasmenyl phosphatidylcholine
plasmenyl-PE: plasmenyl phosphatidylethanolamine
PUFA: Polyunsaturated fatty acid
SM: Sphingomyelin
SOFA: Sequential organ failure assessment
TG: Triacylglycerol
