# Supplementary material for: Associations of the plasma lipidome with mortality in the acute respiratory distress syndrome: a longitudinal cohort study
Source: Respir Res. 2018 Apr 10;19:60. doi: 10.1186/s12931-018-0758-3 (PMC5894233; doi:10.1186/s12931-018-0758-3)
Supplement: Supplementary file 1 — Table S1. Relative lipid concentrations ranked according to the difference between cohorts. (DOCX 52 kb) [file 12931_2018_758_MOESM1_ESM.docx]

# Table S1. Relative lipid concentrations ranked according to the difference between cohorts.

| Lipid* | Survivors | Nonsurvivors | Difference | p-value | q-value† |
| --- | --- | --- | --- | --- | --- |
| PE 32:1 | 0.2477 | 0.5203 | -0.2726 | 0.0017 | 0.0123 |
| PC 31:1 | 0.1896 | 0.4542 | -0.2646 | 0.0000 | 0.0003 |
| PC 37:0 | 0.6966 | 0.9402 | -0.2436 | 0.0124 | 0.0495 |
| PC 31:0 | 0.2242 | 0.4602 | -0.2360 | 0.0002 | 0.0028 |
| PE 38:2 | 0.2436 | 0.4589 | -0.2154 | 0.0013 | 0.0107 |
| plasmenyl PC 44:4 | 0.0076 | 0.0057 | 0.0019 | 0.0121 | 0.0489 |
| DG 38:5 | 0.0138 | 0.0079 | 0.0058 | 0.0006 | 0.0059 |
| PC 37:4 | 0.0257 | 0.0188 | 0.0069 | 0.0068 | 0.0332 |
| SM 43:1 | 0.0186 | 0.0115 | 0.0070 | 0.0001 | 0.0011 |
| lysoPE 22:6 | 0.0193 | 0.0100 | 0.0093 | 0.0039 | 0.0227 |
| plasmenyl PE 40:6 | 0.0208 | 0.0113 | 0.0095 | 0.0026 | 0.0170 |
| DG 38:6 | 0.0164 | 0.0056 | 0.0108 | 0.0000 | 0.0001 |
| TG 58:6 | 0.0336 | 0.0206 | 0.0130 | 0.0026 | 0.0170 |
| lysoPE 20:4_ | 0.0361 | 0.0223 | 0.0139 | 0.0086 | 0.0389 |
| SM 41:1 | 0.0542 | 0.0402 | 0.0141 | 0.0022 | 0.0156 |
| plasmenyl PE 40:5 | 0.0387 | 0.0241 | 0.0146 | 0.0110 | 0.0467 |
| SM 38:4 | 0.0300 | 0.0149 | 0.0150 | 0.0001 | 0.0015 |
| plasmenyl PE 36:4_ | 0.0478 | 0.0322 | 0.0156 | 0.0106 | 0.0459 |
| CE 22:6 | 0.0289 | 0.0119 | 0.0170 | 0.0115 | 0.0478 |
| PE 37:4 | 0.0468 | 0.0293 | 0.0175 | 0.0072 | 0.0348 |
| PE 40:7 | 0.0571 | 0.0374 | 0.0197 | 0.0075 | 0.0353 |
| PE 40:6_ | 0.0472 | 0.0253 | 0.0219 | 0.0023 | 0.0158 |
| SM 39:1 | 0.0531 | 0.0299 | 0.0232 | 0.0030 | 0.0189 |
| SM 42:4 | 0.1021 | 0.0726 | 0.0295 | 0.0081 | 0.0376 |
| TG 58:9 | 0.0449 | 0.0110 | 0.0339 | 0.0000 | 0.0001 |
| SM 40:1 | 0.1395 | 0.0932 | 0.0463 | 0.0014 | 0.0108 |
| PC 40:4 | 0.1638 | 0.1139 | 0.0499 | 0.0101 | 0.0441 |
| TG 51:4 | 0.0859 | 0.0352 | 0.0507 | 0.0000 | 0.0001 |
| CL 70:3 | 0.1831 | 0.1255 | 0.0575 | 0.0116 | 0.0478 |
| PC 40:6 | 0.1398 | 0.0822 | 0.0576 | 0.0000 | 0.0006 |
| TG 56:9 | 0.1045 | 0.0447 | 0.0599 | 0.0000 | 0.0006 |
| SM 42:1 | 0.1987 | 0.1382 | 0.0605 | 0.0073 | 0.0350 |
| PC 34:4 | 0.1713 | 0.1042 | 0.0671 | 0.0046 | 0.0251 |
| PE 34:2 | 0.2177 | 0.1445 | 0.0733 | 0.0082 | 0.0379 |
| DG 36:5 | 0.1302 | 0.0441 | 0.0861 | 0.0000 | 0.0001 |
| PC 33:2 | 0.2175 | 0.1296 | 0.0879 | 0.0002 | 0.0027 |
| PC 35:2 | 0.3902 | 0.3013 | 0.0888 | 0.0060 | 0.0297 |
| SM 37:2 | 0.3240 | 0.2277 | 0.0963 | 0.0119 | 0.0485 |
| DG 40:7 | 0.2100 | 0.1070 | 0.1030 | 0.0000 | 0.0001 |
| SM 38:1 | 0.3796 | 0.2748 | 0.1048 | 0.0039 | 0.0227 |
| TG 50:5 | 0.1767 | 0.0620 | 0.1147 | 0.0000 | 0.00003 |
| TG 48:3 | 0.4134 | 0.2920 | 0.1214 | 0.0041 | 0.0228 |
| PC 36:5 | 0.2935 | 0.1671 | 0.1264 | 0.0011 | 0.0091 |
| PA 34:0 | 0.4033 | 0.2760 | 0.1273 | 0.0090 | 0.0402 |
| plasmenyl PE 36:4 | 0.3927 | 0.2554 | 0.1373 | 0.0052 | 0.0271 |
| TG 56:8 | 0.1947 | 0.0522 | 0.1425 | 0.0000 | 0.00003 |
| PC 38:5 | 0.4398 | 0.2955 | 0.1444 | 0.0006 | 0.0058 |
| PE 40:6 | 0.4057 | 0.2595 | 0.1462 | 0.0007 | 0.0061 |
| TG 56:6 | 0.2725 | 0.1019 | 0.1706 | 0.0000 | 0.0001 |
| SM 36:0 | 0.4668 | 0.2924 | 0.1744 | 0.0021 | 0.0152 |
| CL 78:7 | 0.4642 | 0.2864 | 0.1778 | 0.0040 | 0.0227 |
| TG 58:5 | 0.4756 | 0.2668 | 0.2088 | 0.0005 | 0.0049 |
| lysoPE 22:6_ | 0.3772 | 0.1592 | 0.2180 | 0.0006 | 0.0056 |
| CL 78.:5 | 0.5657 | 0.3298 | 0.2359 | 0.0054 | 0.0278 |
| TG 52:6 | 0.3204 | 0.0827 | 0.2376 | 0.0000 | 0.000003 |
| PE 36:4 | 0.4829 | 0.2363 | 0.2467 | 0.0048 | 0.0257 |
| PC 36:4 | 0.7358 | 0.4844 | 0.2514 | 0.0015 | 0.0112 |
| CL 78:9 | 0.5604 | 0.3043 | 0.2561 | 0.0035 | 0.0213 |
| TG 56:7 | 0.4649 | 0.1855 | 0.2794 | 0.0000 | 0.0001 |
| plasmenyl PE 38:6 | 0.6751 | 0.3740 | 0.3011 | 0.0003 | 0.0035 |
| PE 38:5 | 0.6191 | 0.3117 | 0.3074 | 0.0027 | 0.0171 |
| lysoPE 20:4 | 0.5754 | 0.2582 | 0.3173 | 0.0038 | 0.0227 |
| TG 55:7 | 0.6897 | 0.3262 | 0.3635 | 0.0000 | 0.0003 |
| DG 34:3 | 0.9160 | 0.5254 | 0.3906 | 0.0001 | 0.0021 |
| PC 34:3 | 1.5618 | 1.1156 | 0.4462 | 0.0092 | 0.0409 |
| PC 40:5 | 2.1598 | 1.6898 | 0.4699 | 0.0111 | 0.0467 |
| TG 50:4 | 0.9550 | 0.4519 | 0.5031 | 0.0000 | 0.0001 |
| TG 54:7 | 0.9411 | 0.2537 | 0.6874 | 0.0000 | 0.0001 |
| PC 38:6 | 1.7246 | 0.8668 | 0.8577 | 0.0000 | 0.0001 |
| PE 36:3 | 2.0120 | 1.1312 | 0.8808 | 0.0004 | 0.0044 |
| PA 41:4 | 1.7420 | 0.8588 | 0.8832 | 0.0005 | 0.0049 |
| PC 36:4_ | 2.0056 | 1.0620 | 0.9436 | 0.0001 | 0.0009 |
| TG 56:4 | 2.9610 | 1.9109 | 1.0500 | 0.0051 | 0.0268 |
| TG 54:5 | 3.5883 | 2.5070 | 1.0814 | 0.0005 | 0.0049 |
| TG 52:5 | 1.9578 | 0.6707 | 1.2872 | 0.0000 | 0.00002 |
| TG 53:4 | 2.6435 | 1.3303 | 1.3132 | 0.0000 | 0.0007 |
| TG 52:4 | 8.9955 | 7.6400 | 1.3556 | 0.0026 | 0.0170 |
| plasmenyl PE 34:0 | 2.8575 | 1.4343 | 1.4232 | 0.0015 | 0.0114 |
| PE 38:4 | 2.9383 | 1.4945 | 1.4438 | 0.0013 | 0.0106 |
| SM 33:1 | 6.1937 | 4.6093 | 1.5844 | 0.0060 | 0.0297 |
| DG 36:3 | 6.0699 | 4.2579 | 1.8120 | 0.0012 | 0.0101 |
| CL 82:11 | 6.9315 | 4.9663 | 1.9653 | 0.0033 | 0.0207 |
| TG 54:6 | 3.5610 | 1.5811 | 1.9798 | 0.0000 | 0.0006 |
| TG 54:4 | 5.1874 | 3.1303 | 2.0571 | 0.0003 | 0.0030 |
| PC 36:2 | 14.3677 | 10.9288 | 3.4388 | 0.0043 | 0.0240 |
| DG 36:4 | 6.3417 | 2.2584 | 4.0833 | 0.0000 | 0.0001 |
| PC 34:2 | 14.4732 | 9.8807 | 4.5925 | 0.0002 | 0.0027 |
| CL 78:11 | 18.6118 | 9.9272 | 8.6846 | 0.0008 | 0.0073 |
| PE 38:6 | 21.6334 | 9.9078 | 11.7256 | 0.0001 | 0.0020 |
| TG 52:3 | 224.4894 | 143.3586 | 81.1308 | 0.0005 | 0.0050 |

*See Table 1 for lipid class abbreviations

†p-value adjusted for the false discovery rate
