# Supplementary figures and images for: Associations of the plasma lipidome with mortality in the acute respiratory distress syndrome: a longitudinal cohort study
Source: Respir Res. 2018 Apr 10;19:60. doi: 10.1186/s12931-018-0758-3 (PMC5894233; doi:10.1186/s12931-018-0758-3)

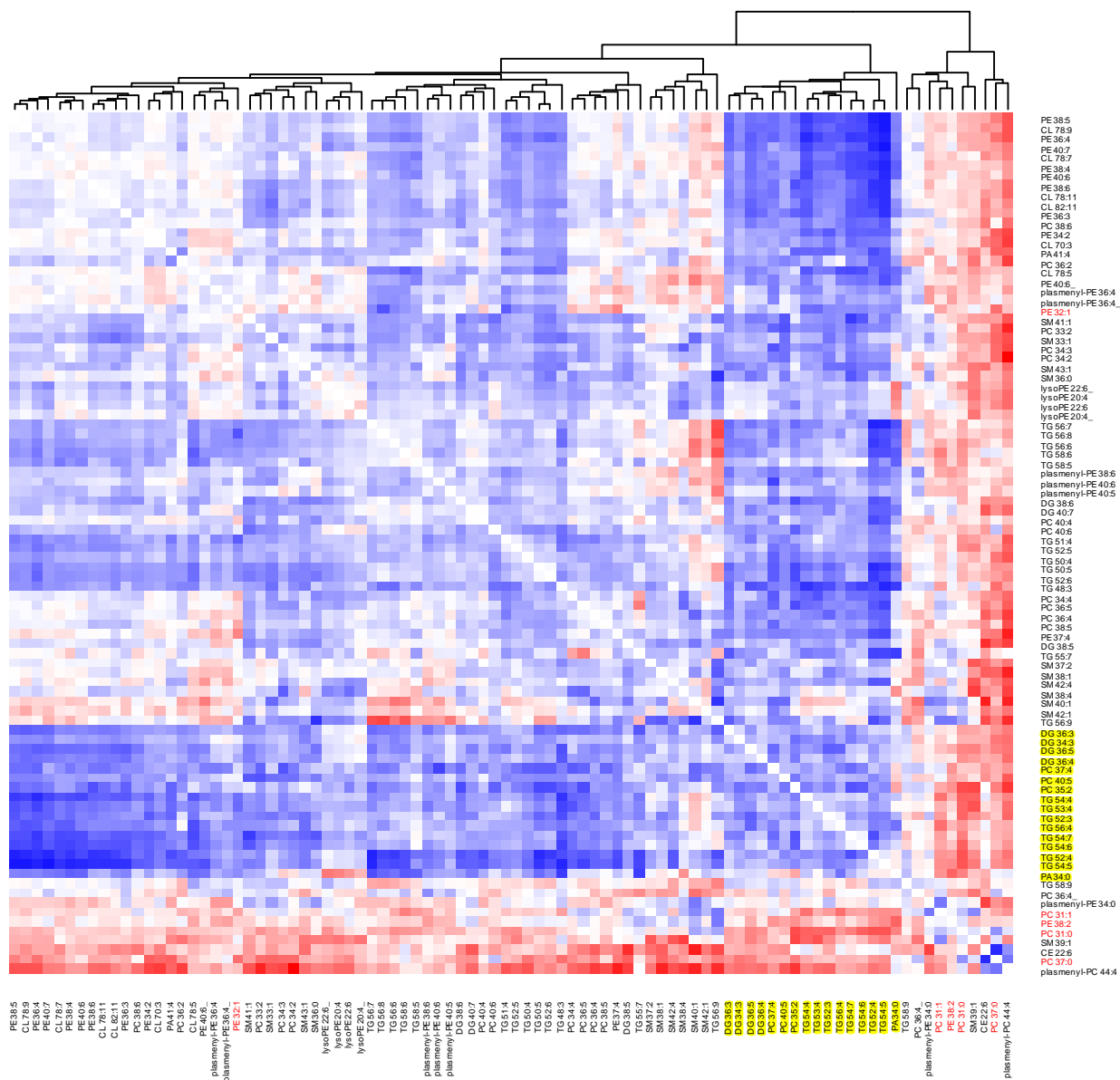

Supplement: Supplementary file 2 — Heatmaps of the differences in the Pearson’s r correlation coefficient between survivors and nonsurvivors. Lipids are arranged based on hierarchical clustering using a complete linkage algorithm. The dendrogram shows how lipids were arranged relative each other. The group of lipids that has a greater correlation in survivors (red font) contains most of the lipids that had a greater concentration in those who died and contains many lipids that originate from the cell membrane. The cluster with the greatest amount of correlation among non-survivors (yellow highlighting) contains primarily glycerolipids consisting mostly of polyunsaturated fatty acids. These observations suggest that these groups of lipids play an important role for the outcome of patients with ARDS. (PDF 119 kb) [file 12931_2018_758_MOESM2_ESM.pdf]
