# Supplementary material for: Associations of the plasma lipidome with mortality in the acute respiratory distress syndrome: a longitudinal cohort study
Source: Respir Res. 2018 Apr 10;19:60. doi: 10.1186/s12931-018-0758-3 (PMC5894233; doi:10.1186/s12931-018-0758-3)
Supplement: Supplementary file 3 — Table S2. Area under the receiver operating characteristic curve for lipids that differ between survivors and non-survivors. (DOCX 35 kb) [file 12931_2018_758_MOESM3_ESM.docx]

# Supplemental Table 2. Area under the receiver operating characteristic curve for lipids that differ between survivors and non-survivors.

| Lipid* | | AUROC | AUROC SD | AOROC 95% CI |
| --- | --- | --- | --- | --- |
| SM 43:1 | | 0.92 | 0.05 | 0.81-1.03 |
| TG 56:6 | | 0.90 | 0.07 | 0.77-1.04 |
| TG 52:6 | | 0.90 | 0.06 | 0.78-1.03 |
| TG 52:5 | | 0.90 | 0.07 | 0.77-1.03 |
| PA 41:4 | | 0.89 | 0.06 | 0.77-1.00 |
| TG 58:9 | | 0.89 | 0.07 | 0.75-1.02 |
| TG 51:4 | | 0.88 | 0.07 | 0.73-1.02 |
| SM 38:4 | | 0.87 | 0.08 | 0.71-1.02 |
| PC 37:0 | | 0.87 | 0.07 | 0.74-1.00 |
| TG 56:8 | | 0.86 | 0.09 | 0.68-1.03 |
| TG 54:7 | | 0.86 | 0.10 | 0.66-1.06 |
| TG 54:6 | | 0.85 | 0.11 | 0.64-1.06 |
| DG 40:7 | | 0.85 | 0.12 | 0.60-1.09 |
| DG 36:5 | | 0.84 | 0.10 | 0.65-1.03 |
| TG 50:5 | | 0.84 | 0.10 | 0.65-1.03 |
| TG 52:3 | | 0.84 | 0.10 | 0.64-1.04 |
| PC 31:1 | | 0.84 | 0.07 | 0.70-0.98 |
| PC 38:6 | | 0.84 | 0.12 | 0.60-1.07 |
| DG 36:4 | | 0.84 | 0.11 | 0.62-1.05 |
| TG 56:7 | | 0.84 | 0.11 | 0.62-1.05 |
| SM 42:4 | | 0.83 | 0.08 | 0.67-0.99 |
| lysoPE 22:6_ | | 0.83 | 0.08 | 0.66-0.99 |
| SM 36:0 | | 0.83 | 0.12 | 0.59-1.06 |
| PC 36:2 | | 0.82 | 0.08 | 0.67-0.98 |
| PC 36:4_ | | 0.82 | 0.09 | 0.65-0.99 |
| TG 53:4 | | 0.82 | 0.10 | 0.63-1.01 |
| TG 50:4 | | 0.82 | 0.11 | 0.61-1.03 |
| TG 54:4 | | 0.82 | 0.09 | 0.64-1.00 |
| SM 37:2 | | 0.81 | 0.09 | 0.64-0.98 |
| DG 38:6 | | 0.81 | 0.12 | 0.58-1.04 |
| TG 54:5 | | 0.81 | 0.12 | 0.57-1.04 |
| PC 34:2 | | 0.80 | 0.11 | 0.59-1.00 |
| PC 40:6 | | 0.80 | 0.11 | 0.59-1.00 |
| lysoPE.20:4 | | 0.79 | 0.09 | 0.61-0.97 |
| TG 52:4 | | 0.79 | 0.13 | 0.54-1.04 |
| PE 38:2 | | 0.78 | 0.09 | 0.61-0.96 |
| PE 38:4 | | 0.78 | 0.10 | 0.58-0.98 |
| TG 55:7 | | 0.78 | 0.10 | 0.58-0.97 |
| PC 31:0 | | 0.78 | 0.08 | 0.61-0.94 |
| PE 36:3 | | 0.77 | 0.12 | 0.53-1.01 |
| DG 34:3 | | 0.77 | 0.13 | 0.51-1.03 |
| PE 34:2 | | 0.76 | 0.10 | 0.57-0.96 |
| CL 78:5 | | 0.76 | 0.11 | 0.54-0.97 |
| PE 38.6 | | 0.76 | 0.12 | 0.53-0.98 |
| PE 38.5 | | 0.75 | 0.10 | 0.55-0.95 |
| plasmenyl PE 38:6 | | 0.75 | 0.13 | 0.49-1.01 |
| PE 36:4 | | 0.74 | 0.10 | 0.54-0.95 |
| CE 22:6 | | 0.74 | 0.10 | 0.55-0.93 |
| PC 33:2 | | 0.74 | 0.12 | 0.49-0.98 |
| plasmenyl PE 40:6 | | 0.74 | 0.12 | 0.50-0.98 |
| plasmenyl PE 34:0 | | 0.73 | 0.12 | 0.49-0.97 |
| CL 82:11 | | 0.73 | 0.13 | 0.49-0.98 |
| CL 78:11 | | 0.73 | 0.12 | 0.50-0.97 |
| CL 78:7 | | 0.73 | 0.12 | 0.49-0.98 |
| lysoPE 20:4_ | | 0.73 | 0.12 | 0.50-0.96 |
| SM 38:1 | | 0.73 | 0.11 | 0.52-0.94 |
| CL 70.:3 | | 0.73 | 0.12 | 0.49-0.97 |
| PE 40:6 | | 0.73 | 0.13 | 0.47-0.98 |
| DG 38:5 | | 0.73 | 0.13 | 0.47-0.99 |
| PC 35:2 | | 0.73 | 0.10 | 0.53-0.92 |
| SM 33:1 | | 0.73 | 0.13 | 0.48-0.98 |
| TG 48:3 | | 0.72 | 0.13 | 0.46-0.98 |
| TG 58:6 | | 0.72 | 0.11 | 0.51-0.93 |
| PC 40:4 | | 0.72 | 0.11 | 0.51-0.93 |
| CL 78:9 | | 0.72 | 0.11 | 0.49-0.94 |
| SM 41:1 | | 0.72 | 0.13 | 0.45-0.98 |
| SM 40:1 | | 0.72 | 0.10 | 0.52-0.91 |
| SM 39:1 | | 0.72 | 0.10 | 0.52-0.91 |
| lysoPE 22:6 | | 0.71 | 0.11 | 0.50-0.92 |
| DG 36:3 | | 0.71 | 0.15 | 0.41-1.01 |
| PC 34:3 | | 0.70 | 0.12 | 0.48-0.93 |
| PE 40:6_ | | 0.70 | 0.12 | 0.47-0.93 |
| PA 34:0 | | 0.70 | 0.14 | 0.42-0.98 |
| plasmenyl PE 40:5 | | 0.69 | 0.13 | 0.44-0.94 |
| TG 56:4 | | 0.69 | 0.12 | 0.46-0.93 |
| TG 56:9 | | 0.69 | 0.13 | 0.43-0.94 |
| TG 58:5 | | 0.69 | 0.12 | 0.45-0.92 |
| plasmenyl PE 36:4 | | 0.68 | 0.13 | 0.42-0.95 |
| plasmenyl PC 44:4 | | 0.68 | 0.12 | 0.44-0.92 |
| SM 42:1 | | 0.68 | 0.10 | 0.48-0.89 |
| PC 37:4 | | 0.68 | 0.15 | 0.40-0.97 |
| PC 36:4 | | 0.68 | 0.13 | 0.42-0.93 |
| PC 36:5 | | 0.67 | 0.13 | 0.42-0.92 |
| APS at randomization† | 0.67 | 0.15 | 0.38-0.96 |  |
| plasmenyl PE 36:4_ | | 0.66 | 0.14 | 0.39-0.93 |
| PC 38:5 | | 0.66 | 0.13 | 0.41-0.92 |
| PE 37:4 | | 0.66 | 0.11 | 0.44-0.89 |
| PE 32:1 | | 0.66 | 0.13 | 0.41-0.92 |
| SOFA† | | 0.64 | 0.12 | 0.41-0.88 |
| PE 40:7 | | 0.64 | 0.13 | 0.38-0.90 |
| PC 34:4 | | 0.64 | 0.13 | 0.37-0.90 |
| PC 40:5 | | 0.64 | 0.14 | 0.36-0.91 |

APS = Acute Physiology Score, AUROC = Area Under the Receiver Operating Characteristic, SD = Standard Deviation, SOFA = Sequential Organ Failure Assessment

*See Table 1 for lipid class abbreviations

†Yellow shading denotes non-lipid variables
